# Cancer risk following organ transplantation: a nationwide cohort study in Sweden

**DOI:** 10.1038/sj.bjc.6601219

**Published:** 2003-09-30

**Authors:** J Adami, H Gäbel, B Lindelöf, K Ekström, B Rydh, B Glimelius, A Ekbom, H-O Adami, F Granath

**Affiliations:** 1 Department of Medical Epidemiology and Biostatistics, Karolinska Institutet, SE-171 77 Stockholm, Sweden; 2 The Transplant Unit, National Board of Health and Welfare, SE-106 30 Stockholm, Sweden; 3 Department of Dermatology, Karolinska Hospital, SE-171 76 Stockholm, Sweden; 4 Department of Oncology, Radiology and Clinical Immunology, University Hospital, SE-751 85 Uppsala; 5 Radiumhemmet, Karolinska Hospital, SE-171 76 Stockholm, Sweden

**Keywords:** organ transplantation, epidemiology, cohort study

## Abstract

A substantial excess risk of lymphomas and nonmelanoma skin cancer has been demonstrated following organ transplantation. Large sample size and long follow-up time may, however, allow more accurate risk estimates and detailed understanding of long-term cancer risk. The objective of the study was to assess the risk of cancer following organ transplantation. A nationwide cohort study comprising 5931 patients who underwent transplantation of kidney, liver or other organs during 1970–1997 in Sweden was conducted. Complete follow-up was accomplished through linkage to nationwide databases. We used comparisons with the entire Swedish population to calculate standardised incidence ratios (SIRs), and Poisson regression for multivariate internal analyses of relative risks (RRs) with 95% confidence intervals (CI). Overall, we observed 692 incident first cancers *vs* 171 expected (SIR 4.0; 95% CI 3.7–4.4). We confirmed marked excesses of nonmelanoma skin cancer (SIR 56.2; 95% CI 49.8–63.2), lip cancer (SIR 53.3; 95% CI 38.0–72.5) and of non-Hodgkin's lymphoma (NHL) (SIR 6.0; 95% CI 4.4–8.0). Compared with patients who underwent kidney transplantation, those who received other organs were at substantially higher risk of NHL (RR 8.4; 95% CI 4.3–16). Besides, we found, significantly, about 20-fold excess risk of cancer of the vulva and vagina, 10-fold of anal cancer, and five-fold of oral cavity and kidney cancer, as well as two- to four-fold excesses of cancer in the oesophagus, stomach, large bowel, urinary bladder, lung and thyroid gland. In conclusion, organ transplantation entails a persistent, about four-fold increased overall cancer risk. The complex pattern of excess risk at many sites challenges current understanding of oncogenic infections that might become activated by immunologic alterations.

Previous epidemiological studies have reported an overall three–five-fold increased risk of neoplasias among transplanted patients compared with the general population (Hoover and Fraumeni, 1973; Kinlen *et al*, 1979; Birkeland *et al*, 1995,^2000^; Hoshida *et al*, 1997; Kyllonen *et al*, 2000; Lindelof *et al*, 2000). The malignancies most consistently associated with organ transplantation include nonmelanoma skin cancer and lymphoproliferative malignancies (Hoover and Fraumeni, 1973; Kinlen *et al*, 1979; Blohme and Brynger, 1985; Barr *et al*, 1989; Kinlen, 1992; Opelz and Henderson, 1993; Birkeland *et al*, 1995, 2000; Bouwes Bavinck *et al*, 1997; Hoshida *et al*, 1997; Kyllonen *et al*, 2000; Lindelof *et al*, 2000; Penn, 2000). There is limited and somewhat conflicting evidence of any relationship between other cancers and transplantation (Hoover and Fraumeni, 1973; Porreco *et al*, 1975; Kinlen *et al*, 1979; Blohme and Brynger, 1985; Barr *et al*, 1989; Kinlen, 1992; Opelz and Henderson, 1993; Penn, 1993,^2000^; Birkeland *et al*, 1995,^2000^; Bouwes Bavinck *et al*, 1997; Hoshida *et al*, 1997; Kyllonen *et al*, 2000; Lindelof *et al*, 2000). Several previous studies were hampered by small size and short follow-up times resulting in lack of statistical precision. Moreover, only few studies (Hoover and Fraumeni, 1973; Kinlen *et al*, 1979; Birkeland *et al*, 1995,^2000^; Hoshida *et al*, 1997; Kyllonen *et al*, 2000) were population based, allowing valid comparisons with the reference population from which the cancer cases arose. In an attempt to further qualify and quantify the risk of cancer following organ replacement, we expanded a previously analysed (Lindelof *et al*, 2000) large population-based nationwide cohort study in Sweden with virtually complete follow-up.

## MATERIAL AND METHODS

This study was approved by the Ethical Committee at the Karolinska Institutet, Stockholm, Sweden.

### The Swedish in-patient register

Since 1965, the National Board of Health and Welfare has collected data on individual hospital discharges in the in-patient register, described in detail elsewhere (Nyren *et al*, 1995). The proportion of the Swedish population covered by this register increased from 60% in 1969 to 85% in 1983, and 100% from 1987 onwards. Each record contains up to eight discharge diagnoses, coded according to the seventh revision of the International Classification of Diseases (ICD-7) 1964–1968, the eighth revision (ICD-8) 1969–1986 and the ninth revision (ICD-9) thereafter. Furthermore, each record contains up to 10 surgical codes, assigned according to the Swedish Classification of Operations and Major Procedures (Nyren *et al*, 1995).

The individually unique 10 digit national registration number ascribed to every Swedish citizen ensures accurate identification and follow-up of each patient who has undergone organ transplantation. When the in-patient register was evaluated for validity and completeness, codes for surgical procedures were correct for 98% of the records (Nilsson *et al*, 1994).

### The Swedish Cancer Registry

The Swedish Cancer Registry, started in 1958, receives reports of all incident malignant tumours diagnosed in Sweden, but does not take into account information from death certificates (The Swedish Cancer Registry, 2001). Reporting by both diagnosing physicians and pathologists is mandatory by law, resulting in registration of more than 98% of all incident tumours, with histological verification of 97% of the tumours. During the years of follow-up for this study, the ICD-7 was used to classify all incident cancers as specified in Table 2. As chronic lymphocytic leukaemias (ICD-7: 204.1) belong to the non-Hodgkin's lymphomas (Harris *et al*, 1994), they were included in this group. The nonmelanoma skin cancer group (ICD-7: 191) does not include basal cell carcinomas since these are not reported to the Swedish Cancer Register.

### Definition of study cohort

**Figure 1:**
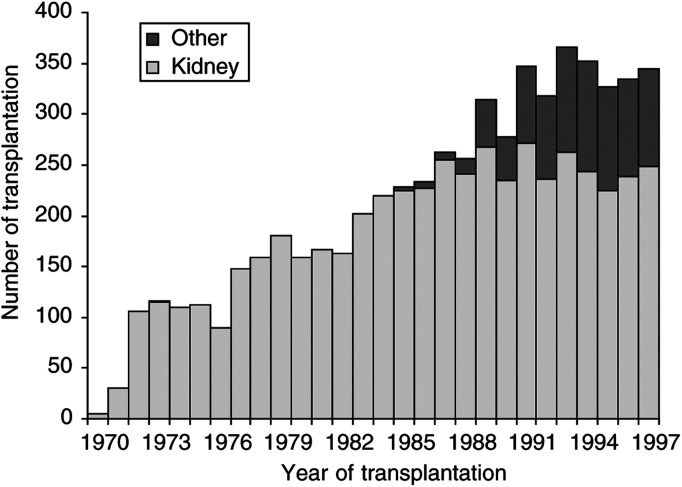
Total number of patients undergoing organ transplantation in Sweden per year during 1970–1997. The dark bars show number of transplantations other than kidney.

In Sweden, a few kidney transplantations were accomplished during the late 1960 s but this activity first became routine during the 1970s (Figure 1

). Organ transplantation is only performed at four specialised centres belonging to public university hospitals; as a result, private in-patient treatment is nonexistent. Therefore, hospital-provided medical services associated with organ transplantation are, in effect, population based. From the in-patient register, we selected all 6457 patients who were hospitalised during 1970–1997 for organ transplantation. Two of four centres (Stockholm and Gothenburg) were not covered by the in-patient registry until 1972 and onwards. Some 50 organ transplantations performed at these centres during 1970 and 1971 were therefore not included in our study cohort (Lindelof *et al*, 2000) unless they were retransplanted after 1971; if so, the date of retransplantation would be erroneously recorded as the first transplantation. The surgical codes used for selection were the following: 6070 (kidney), 5530, 5531 (pancreas), 5200, 5202 (liver), 3590 (lung) and 3085 (heart).

The date of first recorded transplantation marked entry into the cohort. We excluded patients with a previous history of any cancer (*n*=77) or cancers reported within 30 days after transplantation (*n*=179), patients with unknown transplantation codes (*n*=258) or mismatching transplantation dates (*n*=12). Following these exclusions, a total of 5931 eligible patients treated with transplantation of the kidney, liver, heart, lung, pancreas or a combination of solid organs were thus available for follow-up and further analyses.

### Follow-up

The in-patient register was used only to characterise patients with organ transplantation at entry into the cohort. Linkage with the Death register, Register of Population changes and Swedish Cancer Register, respectively, provided information on vital status, emigration and cancer incidence in the cohort during follow-up. We had no reliable information about explantation of organs after the index operation. The follow-up began 30 days after transplantation, in order to exclude prevalent cancers, and continued until diagnosis of cancer, date of death or end of follow-up on 31 December, 1997, whichever occurred first.

### Analyses

Expected numbers of cancers were calculated by multiplying the age, gender and calendar-year-specific risk time by the corresponding cancer incidence rates of the general Swedish population. The standardised incidence ratio (SIR), the ratio of the observed to the expected number of incident cancers, was used to estimate the relative risk (RR) of tumours for different categories. We only counted first cancers (since completeness of reporting second primary malignancies has not been validated), and disregarded prevalent cancers detected incidentally at autopsy (because bias would be introduced if autopsy rates differ between patients with organ transplantation and the general population), both in the cohort and in the expected rates. Confidence intervals of standardised incidence ratio were calculated assuming that the observed number of events followed a Poisson distribution (Breslow and Day, 1987). The observed number of all cancers, nonmelanoma skin cancers, lip cancers and non-Hodgkin's lymphomas (NHLs) – stratified according to all the combinations of the variables given in Table 4 were analysed by Poisson regression with the logarithm of the expected number as an offset term. These analyses yield an assessment of the independent effect of each variable adjusting for potential confounding factors of the others. These analyses were used to assess the statistical significance of each variable, and the results are presented as RRs representing standardised incidence ratios.

## RESULTS

### Overall findings

Selected characteristics of the cohort members are shown in Table 1

**Table 1:** Characteristics of 5931 patients who underwent organ transplantation in Sweden 1970–1997

**Characteristics**	**No. of subjects**	**Proportion**	**Person-years**
Total	5931	100	40 360
Transplanted organ			
Kidney	5004	84.3	36 963
Liver	394	6.6	1254
Heart	236	3.9	960
Lung	117	1.9	324
Pancreas	26	0.4	98
Combination of organs	154	2.5	759
			
Age-distribution at entry	**No. of subjects**		**Proportion (%)**
<40	2 262		38
40–49	1 412		24
50–59	1 487		25
60+	770		13
			
Gender			
Males	3 592		61
Females	2 339		39
	**Years**		**Range**
			
Median age at transplantation	46		0–84
Mean follow-up time	6.8		0–26.8

. A total of 3592 male and 2339 female patients were studied. The vast majority received a kidney transplant (84.3%). The median age at transplantation was 46 years and the mean follow-up time was 6.8 years, yielding a total of 40 360 person-years of follow-up. Figure 1 illustrates the number of patients included per calendar year, showing a successive increase from approximately 100 transplantations per year in the early 1970s to 300 during the 1990s. Through 1984, the cohort only included patients receiving kidney transplants, whereas after that an increasing number of other organ transplantations were performed and corresponded to about 30% of all transplants from the early 1990 s.

Table 2

**Table 2:** Cancer risk following organ transplantation in Sweden, 1970–1997

**Cancer site (ICD-7)**	**Observed no. cases**	**SIR**	**95% CI**
Overall	692	4.0	3.7–4.4
Lip (140)	40	53.3	38.0–72.5
Oral cavity (141–144)	11	5.5	2.7–9.8
Pharynx (145–146)	3	3.1	0.6–9.1
Oesophagus (150)	5	3.2	1.1–7.5
Stomach (151)	12	2.3	1.2–4.1
Small intestine (152)	1	1.1	0.0–6.2
Colon (153)	25	2.3	1.5–3.4
Rectal (154.0+154.1)	14	1.9	1.0–3.2
Adenocarcinomas	9	1.4	0.7–2.6
Squamous cell carcinoma	4	10.2	2.8–26.0
Liver, primary (155)	4	1.1	0.3–2.8
Pancreas (157)	4	0.9	0.3–2.3
Nose, middle ear (160)	2	6.8	0.8–24.5
Larynx (161)	3	2.5	0.5–7.3
Lung (162)	24	1.7	1.1–2.5
Mediastinum (164)	1	42.9	1.1–239
Breast (170)	24	1.0	0.6–1.5
Cervix uteri (171)	5	2.0	0.7–4.7
Cervix *in situ*	52	1.3	1.0–1.8
Ovary (175)	9	2.0	0.9–3.8
Vulva and vagina (176)	11	20.9	10.4–37.4
Vulva	9	26.2	12.0–49.8
Vagina	2	16.4	2.0–59.3
Prostate (177)	20	1.1	0.7–1.7
Testis (178)	3	2.3	0.5–6.6
Kidney (180)	28	4.9	3.3–7.1
Bladder (181)	20	2.3	1.4–3.6
Malignant melanoma (190)	14	1.8	1.0–3.0
Nonmelanoma skin cancer (191)	278	56.2	49.8–63.2
Eye (192)	1	2.0	0.0–10.9
Brain (193)	7	1.0	0.4–2.1
Thyroid (194)	6	3.8	1.4–8.2
Connective tissue (197)	3	2.3	0.5–6.7
Non-Hodgkin's lymphoma (200+202+204.1)	45	6.0	4.4–8.0
Hodgkin's disease (201)	2	2.2	0.3–8.1
Multiple myeloma (203)	6	2.7	1.0–5.9
Lymphocytic leukaemia (204)	3	1.8	0.4–5.1
Myeloic leukaemia (205)	5	2.9	0.9–6.7

Observed number of cases, SIR (SIR) and 95% confidence intervals (CI) by cancer site and type.

summarises the SIRs for different cancer sites following organ transplantation. Overall, we observed 692 primary cancers (counting first cancer at each site) *vs* 171 expected, yielding an SIR of 4.0 (95% CI=3.7–4.4). Table 3

**Table 3:** Cancer risk following organ transplantation in Sweden 1970–1997

**Cancer site (ICD-7)**	**Observed no. cases**	**SIR**	**95% CI**
Non-kidney transplants SIR for selected sites			
Lip (140)	1	24.8	0.6–138.6
Stomach (151)	3	12.0	2.4–35.0
Colon (153)	0	0	0.0–5.8
Rectal (154)	2	4.5	0.5–16.2
Kidney (180)	0	0	0.0–11.2
Nonmelanoma skin cancer (191)	11	34.0	17.0–60.6
Non-Hodgkin's lymphoma (200, 204, 204.1)	18	37.3	22.1–59.1
All cancers	53	4.94	3.7–6.4
			
Kidney transplants SIR for selected sites			
Lip (140)	39	54.8	39.0–74.9
Stomach (151)	9	1.8	0.8–3.4
Colon (153)	25	2.4	1.5–3.5
Rectal (154)	12	1.7	0.9–3.0
Kidney (180)	28	5.2	3.4–7.5
Nonmelanoma skin cancer (191)	267	57.7	51.0–65.1
Non-Hodgkin's lymphoma (200, 204, 204.1)	27	3.8	2.5–5.6
All cancers	639	3.9	3.6–4.2

Observed number of cases, SIR (SIR) and 95% confidence intervals (CI) by cancer site, type and transplanted organ.

summarises the SIRs for selected cancers with adequate numbers for analysis stratified by type of transplantation. The overall cancer risk does not differ significantly between the groups. The risk of developing lip- and nonmelanoma skin cancer is notably higher among renal transplant patients, although this comparison is most likely confounded by the shorter follow-up time among nonrenal transplant patients. Multivariate analysis (Table 4

**Table 4:** Multivariate analyses of relative risks for all cancers and selected cancer sites following organ transplantation

	**All cancers**			**Nonmelanoma skin cancer**			**Lip cancer**			**Non-Hodgkin's lymphoma**		
	**Obs**	**RR**	**95% CI**	**Obs**	**RR**	**95% CI**	**Obs**	**RR**	**95% CI**	**Obs**	**RR**	**95% CI**
Follow-up (years)												
<1	93	1	ref	10	1	ref	0	0	na	16	1	ref
1–4	248	0.9	0.7–1.1	86	2.3	1.2–4.5	14	1	ref	11	0.2	0.1–0.5
5–9	200	1.0	0.8–1.3	92	2.8	1.4–5.3	19	1.6	0.8–3.2	11	0.3	0.2–0.8
10+	151	0.9	0.7–1.2	90	2.4	1.2–4.7	7	0.6	0.3–1.6	7	0.3	0.1–0.7
												
Age at transplantation (years)												
<39	155	2.5	2.0–3.1	71	3.8	2.7–5.4	4	1.6	0.5–5.3	9	4.8	1.6–14.7
40–49	158	1.4	1.1–1.8	51	1.5	1.0–2.2	17	2.8	1.2–6.3	14	3.5	1.2–9.8
50–59	229	1.2	0.9–1.4	90	1.4	1.0–1.9	10	1.0	0.9–1.4	17	2.1	0.8–5.9
60+	150	1	ref	66	1	ref	9	1	ref	5	1	ref
												
Gender												
Male	442	1.5	1.3–1.8	194	0.9	0.7–1.2	29	0.5	0.3–1.1	31	1.0	0.5–1.8
Female	250	1	ref	84	1	ref	11	1	ref	14	1	ref
												
Transplanted organ												
Kidney	639	1	ref	267	1	ref	39	1	ref	27	1	ref
Other	53	1.4	1.0–1.8	11	0.7	0.4–1.3	1	0.5	0.1–3.5	18	8.4	4.3–16

**Figure 2:**
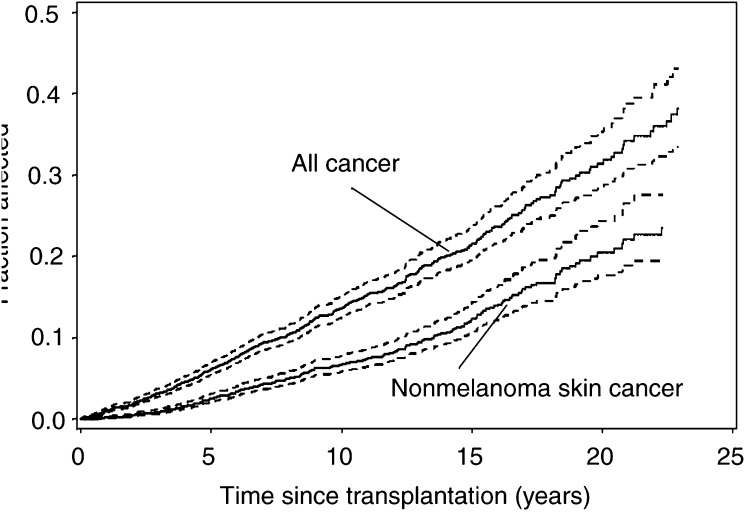
Cumulative risk (solid line) with 95% CI (dotted line) of developing any first malignancy, and nonmelanoma skin cancer, respectively, following transplantation in Sweden, 1970–1997.

Results shown as relative risks (RRs) and 95% confidence intervals (CI) after mutual adjustment for follow-up time, age at transplantation, gender and transplanted organ.

) indicated that this excess risk remained stable during the entire follow-up period. The excess risk decreased monentously with increasing age and was 2.5-fold higher among patients aged less than 40 compared with those who were 60 years or older at transplantation. Relative Risk was also significantly higher in men than in women. Lastly, we compared the overall excess cancer risk in relation to the type of transplanted organ and found slightly lower overall risk following kidney transplantation compared to transplantation of other organ(s) (Table 4).The cumulative risk of developing any malignancy was 13.6% (95% CI=12.4–15.0) 10 years, and 31.8% (95% CI=28.4–35.3) 20 years after transplantation (Figure 2

).

### Head and neck region and gastrointestinal tract

We found a significant almost six-fold excess risk for cancer of the oral cavity based on 11 observed cases (Table 2). The excess was highest for cancer of the tongue (SIR=10.7; 95% CI 4.3–22.1). Based on small numbers, we also found consistent, albeit statistically nonsignificant about three-fold elevated risks for cancer of other parts of the oral cavity and the head and neck region such as salivary glands, floor of the mouth, pharynx and larynx.

A three-fold, significantly increased risk was revealed for oesophageal cancer based on five cases (Table 2). Two of these patients were diagnosed with adenocarcinoma and three with squamous cell carcinomas. Moreover, risk for gastric cancer was increased two-fold.

Although based on small numbers, the risk seemed to be higher for cancer diagnosed in the proximal part of the stomach (SIR=4.4, 95% CI=1.2–11.3) than in the distal parts (SIR=2.2, 95% CI=0.6–5.6).

Similar to the stomach, we found an about two-fold excess risk for cancer of the colon and rectum (Table 2). All colon cancers were adenocarcinomas. The excess risk appeared unequally distributed along the large bowel; SIR for the right-sided colon was 3.3 (95% CI=1.9–5.4), whereas SIR for the left-sided colon (including descending and sigmoid colon) was 1.8 (95% CI=0.7–3.6). For rectal cancer, nine cases were adenocarcinomas (SIR=1.4, 95% CI=0.7–2.6). Four were squamous cell carcinomas, and three of them occurred in women. Since the latter category represents anal cancer, they were analysed separately (SIR=10.2; 95% CI=2.8–26.0). We found no increased risk of cancer of the liver and pancreas (Table 2).

### Urogenital tract

The observed numbers of endometrial (corpus uteri) cancers were close to expected and we found a small statistically non-significant excess of cancers of the cervix and the ovary. Moreover, the incidence of carcinoma *in situ* of the cervix was increased only marginally. In contrast, risk for malignancies in the vulva and vagina were markedly increased (Table 2). Of these cases, nine were vulva (SIR=26.2; 95% CI=12.0–49.8) and two were vaginal cancers (SIR=16.4; 95% CI=2.0–59.3).

There was an almost five-fold increased risk of kidney cancer subsequent to organ transplantation. Of these, 19 originated in the renal parenchyma (SIR=4.1; 95% CI=2.5–6.4), whereas six were of pelvic origin (SIR=10.5; 95% CI=3.9–22.9). An over two-fold statistically significant risk of bladder cancer was revealed; only one of 20 cases was localised in the urethra, whereas the rest occurred in the bladder or at multiple sites. Of the cases, 17 were of transitional cell origin.

### Lip and skin cancer

We found an about 50-fold excess risk of lip cancer (Table 2). Further multivariate analyses revealed no trend with duration of follow-up, but a significantly higher excess among patients younger than 50 years at transplantation compared to those 50 years or older. Women were at higher risk compared to men, although not significantly. We had limited power to detect any difference in risk related to the type of organ transplanted (Table 4).

A markedly increased risk of nonmelanoma skin cancer was found among the cohort members (SIR=56.2, 49.8–63.2), whereas the excess risk of malignant melanoma was modest and statistically not significant (SIR=1.8; 95% CI 1.0–3.0). Multivariate analyses revealed that relative risk for nonmelanoma skin cancer remained relatively constant during 1 or more years of follow-up, and was strongly inversely related to age at transplantation. There was no significant difference between men and women, nor between those undergoing kidney transplantation compared with transplantation with other organs (Table 4). The cumulative risk of developing nonmelanoma skin cancer was 6.7% (95% CI=5.7–7.7) after 10 years and 20.4% (95% CI=17.2–24.6) after 20 years (Figure 2).

### Haematopoeitic and other malignancies

There was a six-fold overall excess risk of NHL following organ transplantation (Table 2). Relative risk was drastically increased during the first year following transplantation (SIR=19.6; 95% CI=11.2–31.9), whereas it remained fairly stable, about four-fold, during all subsequent years. Renal-transplant patients had an increased risk of developing NHL of 9.9 (95% CI 4.0–20.4) during the first year that decreased to 3.2 (95% CI 1.9–4.9) thereafter, whereas nonrenal transplant patients had an increased risk of 38.0 during the first year (95% CI 38.0–157.9) that decreased to 24.3 (95% CI 11.1–46.2) thereafter. Multivariate analyses confirmed this pattern and revealed a strong inverse association with age at transplantation, but no association with gender. Moreover, compared with patients who underwent kidney transplantation, those who received other organs were at eight-fold higher risk after adjustment for follow-up time (Table 4). There was also an indication of excess risks of other haematopoeitic malignancies, although the analyses were based on small numbers (Table 2).

We found an increased risk of lung cancer (SIR=1.7; 95% CI=1.1–2.5). Risk for thyroid cancer was increased four-fold (Table 2) and that for other endocrine tumours was increased eight-fold (data not shown). However, a further analysis of the endocrine group revealed that 31 of the 32 cases were confined to the parathyroid adenomas, presumably reflecting the established association between chronic renal failure and hyperparathyroidism (Gokal, 1988).

## DISCUSSION

Although cancer risk following organ transplantation has been analysed in several prospective studies, only few of them were population based with long-term follow-up (Hoover and Fraumeni, 1973; Kinlen *et al*, 1979; Birkeland *et al*, 1995, 2000; Hoshida *et al*, 1997; Kyllonen *et al*, 2000; Lindelof *et al*, 2000), and except for one of the studies (Lindelof *et al*, 2000), none of them included patients with another transplanted organ than a kidney. While components of our study have been included in earlier analyses (Birkeland *et al*, 1995) and chiefly in a publication focused on skin cancer (Lindelof *et al*, 2000), the cohort has now been expanded and the follow-up time added. This expansion has entailed the largest population-based study so far allowing more precise quantification of risk and also separate analyses of several anatomic and histopathologic subtypes. We also had the possibility at comparing cancer risk following transplantation of a kidney with that of other organs, and to analyse effectively duration of follow-up, age at transplantation and gender as determinants of cancer risk.

Our study confirmed a four-fold overall risk of cancer following organ transplantation and further indicated that this excess risk remains seemingly stable during longer follow-up. Moreover, we confirmed a significantly higher overall risk among patients transplanted at young age, among men compared with women, and following transplantation of organs other than a kidney (Kinlen, 1992; Penn, 2000). For specific cancer sites, the excess risk was most notable for nonmelanoma skin cancer, lip cancer and NHL, which is in accordance with other investigators (Hoover and Fraumeni, 1973; Kinlen *et al*, 1979; Blohme and Brynger, 1985; Barr *et al*, 1989; Kinlen, 1992; Opelz and Heneerson, 1993; Birkeland *et al*, 1995,^2000^; Bouwes Bavinck *et al*, 1997; Hoshida *et al*, 1997; Kyllonen *et al*, 2000; Lindelof *et al*, 2000; Penn, 2000). In the study by Lindelöf *et al* (2000), the SIRs included all incident cancers, whereas in our study, we only counted the first cancer. This possibly explains the differences in RR magnitudes between these two studies. Novel findings in our study include the substantial excess risk for cancer of the tongue, anus, vulva and vagina, but no convincing excess risk for *in situ* or invasive cancer of the cervix as reported previously (Porreco *et al*, 1975; Sillman *et al*, 1984), and evidence of a higher risk for kidney cancer with pelvic origin than that of parenchymal origin.

While we consider the internal validity of our study to be high, several limitations should be emphasised. Since we were unable to scrutinise almost 6000 medical records individually, we had to rely on information from the In-patient Discharge Register. It is difficult to reconstruct from this register accurately the course of events following an organ transplantation which may include explantation as well as one or several replantations. Most likely, neither failure to censor follow-up in some patients because they were neither transplanted nor immunosuppressed entails some underestimation of excess risks. Notwithstanding the relatively large size of the study, we had insufficient power to analyse several rare malignancies and to confirm moderate excess risks. Moreover, we could not effectively adjust for potential confounding because we had neither data on life-style factors among organ-transplanted patients nor among the general Swedish population that generated the expected cancer incidence rates. About 30% of transplanted patients have diabetes (The Swedish Registry of Vrenic Care 2000), a disease associated with an excess risk of several malignancies (Adami *et al*, 1991). Diabetes is unlikely, however, to explain our pattern of findings because this disease is associated most strongly with cancer of the liver (Lagiou *et al*, 2000) and endometrium (Weiderpass *et al*, 2000), two sites for which we observed no excess risk following transplantation. Long-term dialysis preceding kidney transplantation is another conceivable confounder, although studies of cancer risk in such patients have been inconclusive (Kantor *et al*, 1987; Fairley *et al*, 1994; Maisonneuve *et al*, 1999; Birkeland *et al*, 2000). Lastly, many of the excess risks we observed were substantially higher than those generally found in relation to established risk factors for the respective malignancies. Hence, confounding may indeed be a limited concern and most of the risks we observed might be attributable to the organ transplantation and subsequent immunosuppressive therapy.

Our study was not designed to elucidate the numerous mechanisms that have been invoked to explain the excess cancer risk following organ transplantation (Penn, 1991). Among these, immune modulation is the most obvious, and likely the most important. Prevalent infection with several potentially oncogenic viruses and bacteria, such as EBV, human papilloma virus (HPV), HHV-8, HTLV-1, HIV and *Helicobacter pylori*, might become activated by impaired immune function (Goedert, 2000). As several of these, notably HHV-8, HTLV-1 and HIV, are rare in the Swedish population, measurable effects on cancer risk appear unlikely. Although EBV may account for the substantial excess risk of NHL – appearing with a short latency and being most marked after organ transplantation other than the kidney – the possible role of other viruses yet to be detected is presently unknown. Although activation of *H. pylori* might explain the excess risk of stomach cancer, this bacterium appears most strongly associated with distal stomach cancer (Huang *et al*, 1998) while we, if anything found higher excess risk for proximal cancers.

Most enigmatic were our findings with regard to cancers associated with HPV. Unknown HPV subtypes are hypothesised to play a role in the aetiology of nonmelanoma skin cancer (Blohme and Brynger, 1985; Barr *et al*, 1989; Bouwes Bavinck *et al*, 1997) and defined oncogenic types are clearly causally related to cancer of the cervix (IARC, 1995), anus (Frisch *et al*, 1997), vulva and vagina (Blohme and Brynger, 1985; Alloub *et al*, 1989). Although controversial, HPV has also been proposed as a risk factor for oesophageal cancer (Galloway and Daling, 1996) as well as for head and neck cancers (Mork *et al*, 2001). However, if altered immune function entails activation of HPV infection and thereby increased viral load, an excess risk of malignancies of the cervix should be expected (Ylitalo *et al*, 2000), but no convincing excess was observed. This leads us to hypothesise that anatomic differences in immunologic control of HPV-harbouring target cells explains the spectacular differences in risk for HPV-associated malignancies. The substantial excess risk for numerous malignancies without any established or suspected infectious aetiology further suggests that the mechanisms by which organ transplantation increases cancer risk may be more complicated than an infectious-driven malignant transformation. This finding seems to challenge the established concept that most tumours are nonimmunogenic. Moreover, recent experimental data from immunodeficient double-knockout mice show increased incidence of several spontaneous tumour types (Shankaran *et al*, 2001).

In conclusion, we provide further evidence of an association between organ replacement and the development of a number of different malignancies. These results indicate the need for further investigation as the numbers of transplant recipients increase and their life expectancy improves. A better understanding of factors that determine cancer risk might serve several purposes. First, an immunosuppressive regimen could be designed to minimise cancer risk whenever possible. Second, a better quantification of cancer risks might facilitate early diagnoses and thereby potentially improve prognosis. Lastly, detailed studies of cancer following organ transplantation might advance our understanding of the carcinogenic process and the role of immune surveillance for several cancer sites and types.
